# Alignment of European Regulatory and Health Technology Assessments: A Review of Licensed Products for Alzheimer's Disease

**DOI:** 10.3389/fmed.2019.00073

**Published:** 2019-05-07

**Authors:** Marieke J. H. J. Dekker, Jacoline C. Bouvy, Diana O'Rourke, Robin Thompson, Amr Makady, Pall Jonsson, Christine C. Gispen-de Wied

**Affiliations:** ^1^Medicines Evaluation Board, Utrecht, Netherlands; ^2^National Institute for Health and Care Excellence, London, United Kingdom; ^3^National Institute for Health and Care Excellence, Manchester, United Kingdom; ^4^Biogen, Baar, Switzerland; ^5^National Health Care Institute, Diemen, Netherlands

**Keywords:** health technology assessments, regulatory assessments, alignment, Alzheimer's disease, regulatory science

## Abstract

**Aims:** To facilitate regulatory learning, we evaluated similarities and differences in evidence requirements between regulatory and health technology assessment (HTA) bodies of Alzheimer's disease (AD) approved products.

**Methods:** The European marketing authorisation application dossiers and European public assessment reports (EPARs) of the licensed AD drugs were screened to identify the phase III randomised controlled trials (RCTs) and outcomes used. We also screened the assessment reports of the National Institute of Health and Care Excellence (NICE, England) and the National Health Care Institute (ZiN, the Netherlands) to identify the studies and outcomes used in HTA assessments.

**Results:** The application dossiers of donepezil, galantamine, rivastigmine, and memantine contained 16 phase III RCTs in total. These trials were also included in HTA assessments except that NICE excluded studies that were not published (*n* = 2) or trials that included patients with other types of dementia (*n* = 3). In the regulatory assessments the focus was on cognitive and global outcomes, and to some extent on function. In the HTA assessments of clinical effectiveness other domains were also covered including: function, behaviour and mood, and, occasionally, quality of life. In the economic analyses of NICE the domains cognition, function, and quality of life were included.

**Conclusion:** There was a large overlap in inclusion of trials in regulatory and HTA assessments, although the focus on specific outcomes slightly differed. Understanding the methods and perceptions of both authorities can stimulate regulatory and HTA cross-talk and further alignment, and therefore more rapid patient access to new treatments.

## Introduction

Alzheimer's disease (AD) is the most common cause of dementia worldwide and in Europe; the estimated prevalence of the disease ranges from 1 to 2% in persons aged 60 years and older, and the prevalence of AD is expected to increase substantially in the next decade (1). Currently, two different classes of AD drugs are available on the European market: the cholinesterase inhibitors (rivastigmine, donepezil, and galantamine) and one non-competitive N-methyl-D-aspartate receptor antagonist (NMDA; memantine) (2). As these drugs are symptomatic treatments, providing only temporary and modest improvement in AD symptoms, a large unmet medical need remains in AD. There are now around 100 AD drugs in the drug-development pipeline, of which around 70% are disease-modifying agents (3).

Patient access to new drugs requires marketing authorisation from a regulatory authority and reimbursement by a payer. In the European Union (EU), the centralised marketing authorisation procedure is compulsory for drugs that treat neurodegenerative disorders in humans (4). Under this procedure, the company submits a single marketing authorisation dossier to the European Medicines Agency (EMA) and will be granted a marketing authorisation for the European Economic Area if the drug's benefit-risk profile is positive. Health technology assessments (HTA) and reimbursement decisions take place at a national or regional level and methods vary between regions. The focus is usually on clinical effectiveness, cost-effectiveness and budget impact of a drug as compared to existing treatment options (5, 6).

Due to differences in evidence requirements it can be challenging for pharmaceutical companies to design clinical trials that meet the needs of both regulatory and HTA agencies (7). Several initiatives have been launched in recent years to increase the interaction between medicines' developers, regulators and HTA bodies and the EMA now offers joint scientific advice with the European Network for Health Technology Assessment (EUnetHTA), allowing companies to obtain feedback on their development plans from both regulators and HTA bodies (8).

Narrowing the gap in evidence requirements between regulatory and HTA agencies could facilitate more efficient drug development and earlier patient access to promising treatments. To inform constructive collaborations between all stakeholders it is important to determine what differences in evidence requirements actually exist. Therefore, the objective of this study was to assess differences and similarities in inclusion of clinical trials, outcome domains, and the use of real world evidence in regulatory and HTA assessments of the four currently available AD drugs.

## Methods

We included the three cholinesterase inhibitors (donepezil, galantamine, and rivastigmine) and the NMDA antagonist memantine in this study as they are the currently authorised drugs for AD. Donepezil and galantamine were approved through the European mutual recognition procedures in 1997 (2011 in the Netherlands) and 2000, respectively, and rivastigmine and memantine through the European Medicines Agency's (EMA) centralised procedures in 1998 and 2002.

### Regulatory Assessments

**Figure 1:**
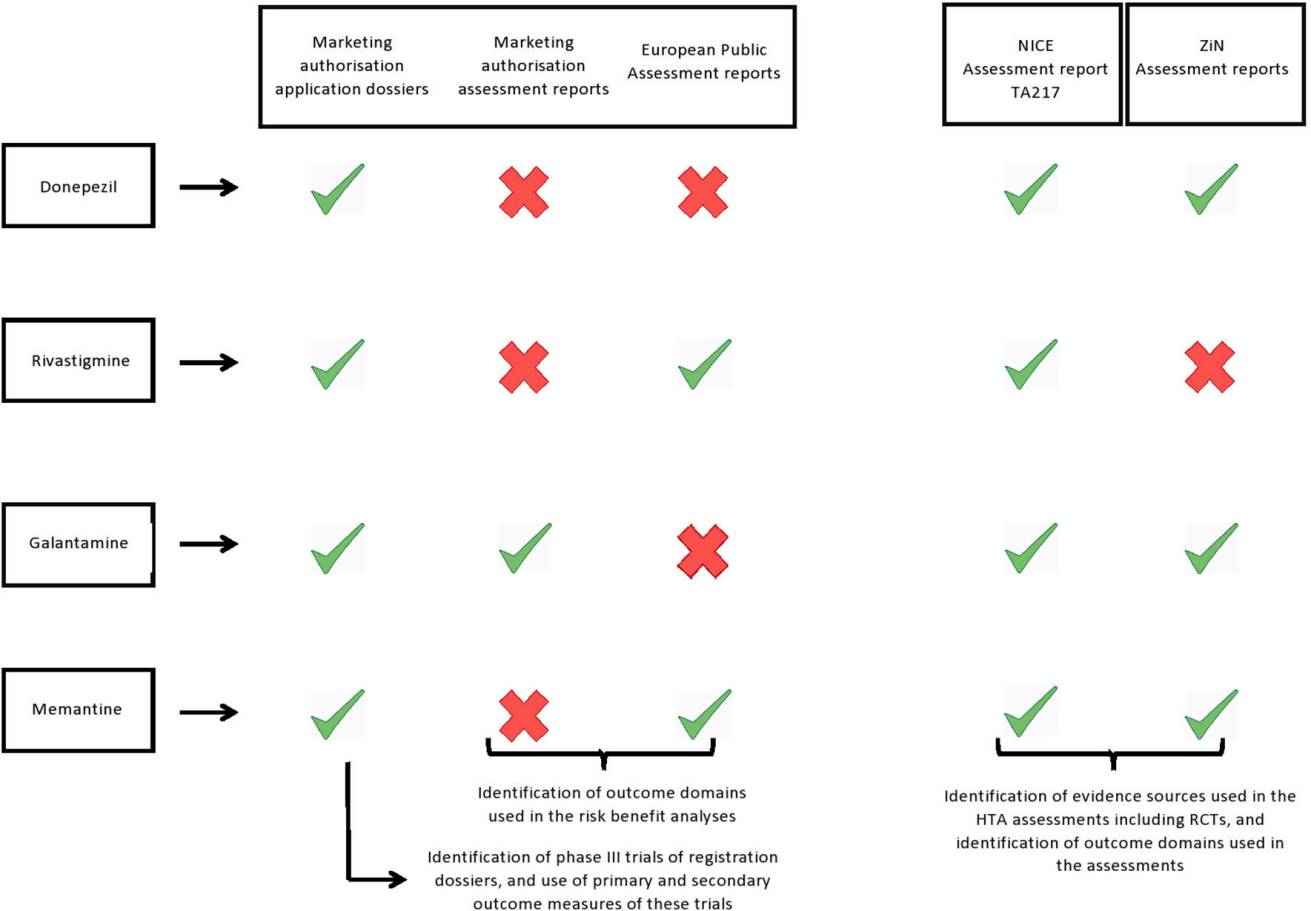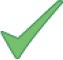 Report was included in our study. 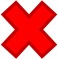 Report was not available and was therefore not included in our study.

We collected all information from the application dossiers for marketing authorisation, regulatory assessment reports, and the European public assessment reports (EPARs) that were accessed through the Dutch Medicines Evaluation Board database (application dossiers for marketing authorisation of the mutual recognition procedures (donepezil and galantamine), the summary of the application dossiers for the centralised procedures (rivastigmine and memantine), and the regulatory assessment report of galantamine), or the EMA website (EPARs, rivastigmine and memantine) (9, 10). See Figure 1 for an overview of all included data sources.

We extracted the following information on the marketing authorisation procedures of AD products: year of approval, product name, and the indication for which a product was approved. Additionally, detailed information was collected from the phase III randomised controlled trials included in the application dossiers: name of the study, study design, drugs and dosages, number of participants, primary and secondary endpoints, and whether the study had been published in a peer reviewed journal. Furthermore, from the EPARs and the regulatory assessment report we extracted which outcome domains were used in the regulatory assessments.

### HTA Assessments

We included the HTA reports from The National Institute of Health and Care Excellence (NICE; England's HTA body) and from the National Health Care Institute (ZiN; Dutch HTA body) as the authors had access to the assessment reports required for this study. NICE's most recent assessment encompassed all four AD drugs and is publically available (Technology Appraisal TA217: Donepezil, galantamine, rivastigmine and memantine for the treatment of Alzheimer's disease). The ZiN assessment report of donepezil was accessed through the ZiN website (11), but the galantamine and memantine reports were not publicly available and were obtained from ZiN directly. In the current study, we included both the first ZiN assessment report of memantine (2003) and the reassessment report of 2004, as memantine was only reimbursed in the Netherlands after the second assessment. We could not access the ZiN assessment report of rivastigmine and therefore it was not included in this study (Figure 1).

We extracted the following data from the HTA reports: publication date, indication(s) for which the product was assessed, overview of included studies, outcome domains of the assessment, and use of real world evidence. For the NICE appraisals we also evaluated the effectiveness measures used in the economic analyses. No economic analyses were used in the available ZiN assessments.

## Results

### Clinical Trials Included in Regulatory and HTA Assessments

We found that out of the 16 phase III trials that were included in the marketing authorisation application dossiers of the four drugs, 12 were included in the ZiN appraisals (no data available for rivastigmine) and 11 were included in the most recent NICE appraisal (Table 1). The reasons for exclusion of five trials in the NICE report were that patients with other types of dementia were included (n = 3) or the results of those trials were not, or only partially, published in peer-reviewed journals (*n* = 2) (Table 2). Supplementary Table 1 gives an overview of the characteristics of all 16 phase III RCTs included in the marketing authorisation application dossiers, including information on the trial arms, number of participants, disease severity of included patients, and primary and secondary outcome measures.

**Table 1:** Alignment of regulatory and HTA assessments of NICE and ZiN.

**AD product**	**Regulatory assessment**	**NICE, TA217**	**ZiN**
Donepezil			
- *Year assessment*	1997	2011	2013
- *Main Sources of evidence*	3 phase III RCTs^a^: - E2020-A0001-302 (12) - E2020-A0001-301 (13) - E2020-A0001-304 (14)	19 RCTs (12–30), including: - E2020-A0001-302 (12) - E2020-A0001-301 (13) - E2020-A0001-304 (14) 7 head-to-head comparisons (31–37)^b^ systematic reviews (38, 39) Submissions of consultees and personal statements^c^	1 Systematic review (40), including: - E2020-A0001-302 (12) - E2020-A0001- - 301 (13) - E2020-A0001-304 (14) - 45 additional RCTs 2 head-to-head comparisons (31, 41) Registration texts^d^
- *Main outcome domains*	Cognition Global effect	Cognition Global Effect Function Behaviour and mood Quality of Life	Cognition Global effect Function Behaviour and mood
Rivastigmine			
- *Year assessment*	1998	2011	*Report not available*
- *Main Sources of evidence*	4 phase III RCTs: - B351 (42) - B352 (43) - B303 (44) - B304 (45)	7 RCTs (43–49), including: - B352 (43) - B303 (44) - B304 (45) 5 head-to-head comparisons (31, 33, 34, 36, 37)^b^ 3 systematic reviews (38, 39, 50) Submissions of consultees and personal statements^c^	
- *Main outcome domains*	Cognition Global effect	Cognition Global Effect Function Behaviour and mood^e^	
Galantamine			
- *Year assessment*	2000	2011	2003
- *Main Sources of evidence*	5 phase III RCTs: - GAL-95-05 - GAL-INT-1 (51) - GAL-INT-2 (52) - GAL-USA-1 (53) - GAL-USA-10 (54)	8 RCTs (51–59), including: - GAL-INT-1 (51) - GAL-INT-2 (52) - GAL-USA-1 (53) - GAL-USA-10 (54) 4 head-to-head comparisons (32, 33, 35, 36)^b^ 2 systematic reviews (38, 39) Submissions of consultees and personal statements^c^	6 RCTs (51–55, 60), including: - GAL-INT-1 (51) - GAL-INT-2 (52 - GAL-USA-1 (53) - GAL-USA-10 (54) 2 systematic reviews (50, 61): (61) included all regulatory phase III trials including GAL-95-05 NICE TA19 (62) Registration texts^d^
- *Main outcome domains*	Cognition Global effect (Function)^f^	Cognition Global Effect Function Behaviour and mood^e^	Cognition Global effect Function
Memantine			
- *Year assessment*	2002	2011^g^	2003 and 2004
- *Main Sources of evidence*	4 phase III RCTs - MRZ-9605 (63) - MRZ-9403 (64) - MRZ-9202 (65) - MRZ-9408 (66)	2 RCTs (63, 67), including: - MRZ-9605 (63) 1 systematic review that included all four AD products (38) Submissions of consultees and personal statements^c^	Registration texts^d^, that included results of: - MRZ-9605 (63) - MRZ-9403 (64) - MRZ-9202 (65) - MRZ-9408 (66) 1 RCT (68) *Post-hoc* analyses on RCT-data (69, 70)
- *Main outcome domains*	Cognition Global effect Function	Cognition Global Effect Function Behaviour and mood^e^	Cognition Global effect Function

a *RCTs randomised controlled trials*.

b *three head-to-head trials (32, 33, 35) were described in detail in the NICE assessment report, however they were considered to be of insufficient quality to inform the review*.

c *consultees include submissions from patient and professional organisations and manufacturers; personal statements include statements from patient and professional experts*.

d *registration texts included EPARs (when available) and summaries of product characteristics (SmPC)*.

e *NICE intended to assess quality of life, but there was not enough randomised controlled evidence available to perform this assessment*.

f *the outcome domain ‘function' was mentioned in the summary of the regulatory assessment report, but it was not mentioned in the section of the risk-benefit analysis*.

g *NICE additionally assessed the effectiveness of memantine in combination with a cholinesterase inhibitor versus cholinesterase inhibitor monotherapy separately. In this assessment 2 RCTs were included (68, 71)*.

**Table 2:** Reason of exclusion of phase 3 trials of the marketing authorisation dossiers in the NICE assessment of clinical effectiveness.

**Drug**	**Trial**	**Reason for exclusion in the NICE assessment**
Galantamine	GAL-95-05	Trial was not published
Rivastigmine	B351, Schneider et al. (36)	In the publication of this trial the results are pooled with the results of two other trials (37, 38) and the results from this individual trial could not be extracted from this publication
Memantine	MRZ-9403, Winblad et al. (57)	Next to patients with probable AD, patients with probable vascular of mixed type dementia were included
	MRZ-9202, Wilcock et al. (58)	Only patients with vascular dementia were included
	MRZ-9408, Orgogozo et al. (59)	Only patients with vascular dementia were included

### Outcome Measures and Domains Included in Regulatory and HTA Assessments

Overall, a large variety of different outcome measures were used in both the HTA assessments and the phase III trials of the marketing authorisation dossiers. Five main outcome domains could be identified: cognition, function, global effect, behaviour and mood, and quality of life. Supplementary Table 2 gives an overview and glossary of the outcome measures used per outcome domain.

We identified slight differences in outcome domains that were included in the regulatory assessments compared with the HTA assessments (Table 1). In the risk-benefit analyses of the regulatory assessments, only primary outcomes of the registration trials were considered; namely cognition and global effect for mild-to-moderate AD (donepezil, galantamine, rivastigmine, and memantine) and function and global effect for moderate-to-severe AD (memantine). However, in the only available regulatory assessment report (galantamine), the results of the secondary outcome measures (function and behaviour and mood) were also mentioned, and thus implicitly assessed. In the assessment of clinical effectiveness, NICE evaluated the totality of evidence, including the outcome domains cognition, function, global effect, behaviour and mood, and quality of life, however, due to a lack of randomised evidence, quality of life could only be assessed for donepezil. In the economic analyses of NICE the domains cognition, function, and health related quality of life were included. In the model, health-related quality of life (EuroQol-5 Dimensions, EQ-5D) was mapped from cognition values.

ZiN assessed the outcome domains cognition, function, and global effect. Additionally “behaviour and mood” was assessed in the report of donepezil.

### Types of Evidence Included in HTA Assessments

The NICE assessment (TA217) evaluated the clinical effectiveness of the AD products by a systematic review of research evidence. Only systematic reviews of RCTs and RCTs were included (Table 1). Where data permitted, the results of the individual trials were pooled using meta-analyses (72). The manufacturers of donepezil submitted a systematic review that included both RCTs and observational studies. Results from observational data were specifically used to support the effectiveness of donepezil beyond 6 months of use, its effect on mortality, impact on care-giver stress and carer-time, and symptoms after withdrawal (72). The other manufacturers did not submit observational data. The submissions of the manufacturers were mainly used to compare their findings with the results of the systematic review of NICE.

In the ZiN assessment of galantamine the main evidence sources were the summary of product characteristics (SmPC) from the registration dossier and two Cochrane reviews of galantamine and rivastigmine (50, 61). In addition, a systematic review that included all three cholinesterase inhibitors (73), six randomised controlled trials (51–55, 60), and the 2001 NICE appraisal (62) were included. In the ZiN assessment report of donepezil (2013), comparators were rivastigmine and galantamine and main evidence sources were SmPC's, two head-to-head trials (31, 41), and one meta-analysis (40). The main source of evidence for the ZiN assessment of memantine in 2003 was the EPAR. In the reassessment of memantine (2004) one additional RCT was included and two publications that included *post-hoc* analyses on RCTs that were already included in the first assessment (68–70). ZiN did not include observational studies or other types of real-world evidence in any of the assessments.

## Discussion

The current study shows that the gap between regulatory and HTA assessments of approved AD drugs was not as large as it might be perceived. There was a large overlap in inclusion of RCTs, although the focus on specific outcomes slightly differed between regulatory and HTA assessments. In the assessment of clinical effectiveness, HTA evaluates the totality of evidence, including the outcome domains cognition, function, global effect, behaviour and mood, and, occasionally, quality of life and observational data. In the economic assessment of NICE only the dimensions cognition, function, and quality of life were included in the model. In contrast, in the risk benefit analyses of the regulatory assessments only primary outcomes of the registration trials were taken into account, namely cognition and global effect for mild-to-moderate AD and function and global effect for moderate-to-severe AD. Secondary endpoints were mentioned in the regulatory assessment report and are thus implicitly assessed as well.

All four drugs included in this study have been available for many years. In September 2018 the new EMA guideline on the clinical investigation of medicines for the treatment of AD came into effect, which distinguishes between patients with established AD, prodromal AD or mild cognitive impairment, and preclinical disease (74). For patients with established AD efficacy should be specified for the domains cognition, function, and global effect, with secondary endpoints including health-related quality of life, and behavioural and psychiatric symptoms. All these outcome domains were part of the HTA assessments included in our study, suggesting that for AD, regulatory and HTA requirements might not be far apart with respect to preferred endpoints.

The majority of AD drugs that are currently in the pipeline are disease-modifying agents that intend to prevent or slow disease progression and usually target underlying pathophysiologic mechanisms (e.g., amyloid and/or tau) early in the disease course (3). The EMA guideline indicates that in earlier disease stages, such as mild cognitive impairment (MCI) and prodromal AD, the use of primary endpoints assessing cognition and function or global might be difficult due to limitations of the currently available instruments. However, it remains important to demonstrate that the effects of treatments are clinically relevant. Possible solutions could be constructing more sensitive scales, investigating in detail only those domains that have been shown to be impaired in the early disease stages or the use of composite scales assessing both cognition and daily functioning as a single primary endpoint (74). Currently, there is no gold standard for the assessment of treatment effects in patients with preclinical AD. Prevention trials require at least large samples and long follow-up until a reliable and meaningful outcome is reached. In the EMA guideline, the main treatment goal remains prevention of cognitive decline. However, since a firm regulatory framework is lacking, no firm recommendation could be made in the guideline and therefore scientific advice is recommended (74). In addition, for the HTA perspective it will be essential to define what a clinically meaningful benefit might be for a disease-modifying drug that prevents or delays cognitive symptoms.

One of the main differences between regulatory and HTA assessments is that the latter will compare new drugs to the standard of care, whereas in regulatory assessments treatments are often compared with placebo. In general, HTA bodies will assess whether the new drug is more effective than the current standard of care, but a marketing authorisation does not require a drug to demonstrate superiority against comparators. In case of the current AD drugs, the standard of care was “best supportive care,” which meant that the RCTs used for marketing approval were also used for HTA appraisals, although the HTA assessments also compared the AD drugs with each other. The relative effectiveness of the different cholinesterase inhibitors was assessed using head-to-head trials or indirect evidence, i.e., RCTs with placebo arms or systematic reviews and meta-analyses.

Absence or poor quality of head-to-head comparisons might impact reimbursement decisions, because indirect comparisons might introduce uncertainty of the added therapeutic value of new treatments. In the evaluated HTA assessments of NICE and ZiN only a few good quality head-to-head trials were available. However, this did not impact reimbursements decisions, since there was sufficient evidence that demonstrated the clinical effectiveness of the different cholinesterase inhibitors was comparable (NICE and ZiN) and all treatments were considered cost-effective (NICE). However, it is important to note that the last NICE appraisal was concluded several years after the introduction of the drugs to the market, and the first HTA of a new, disease-modifying treatment might have to rely on less comprehensive evidence than that was available for this appraisal.

Another difference between regulatory and HTA assessments is that some HTA bodies, like NICE, reassess technologies over time (5). NICE appraised the approved AD drugs in 2001, 2006, and 2011. As more evidence on the effectiveness and safety of the AD drugs had become available, a large number of RCTs, systematic reviews, and head-to-head trials were included in the most recent NICE appraisal. The use of real world evidence was limited, probably due to the availability of a large amount of RCT evidence (72). The role of real world data in both regulatory and HTA assessments may increase once new treatments for AD need to be assessed. Currently, there are no gold standards for efficacy parameters for people with preclinical AD and long term studies will be needed to validate surrogate endpoints and model disease progression, preferably in a real world setting. Also, the latest EMA guideline on good pharmacovigilance practices states that in the post-marketing risk benefit analyses both clinical trial and real world data should be included (75).

Over the past decade, several initiatives have been launched to better align the assessments of regulatory and HTA bodies. In 2010, the EMA started a pilot on parallel regulatory-HTA scientific advice. As of July 2017, this procedure was replaced by the EMA-EUnetHTA Parallel Consultation, which is a single gateway for scientific advice from EMA and HTA bodies (8). In addition, EMA launched PRIME (PRIority MEdicines) in 2016 to promote early access to medicines that offer clear benefit over existing treatments or benefit for patients with no current treatment options (76). PRIME helps medicines developers with their development plans, offers continuous support, provides regulatory guidance, and consults other stakeholders including HTA bodies.

Wang et al. studied the impact of all these initiatives in different regions. They found that all stakeholders confirmed that gaps in evidence requirements between both regulators and HTA bodies had narrowed over the past 5 years (77). However, several divergences with relevance for AD were identified. Conditional or accelerated regulatory approvals were not well-aligned with flexible HTA approaches, possibly hampering early patient access. Furthermore, areas for improvement included acceptable primary and surrogate endpoints, inclusion of an active comparator arm in the trial, definition of unmet medical need, and post-marketing evidence generation.

Although regulatory approval of drugs is harmonised within the European Union, reimbursement decisions are still the responsibility of the individual member states, and different countries use different methods and processes for making these decisions. This can result in different reimbursement recommendations between countries or sometimes even regions when decision making is decentralised (78). In addition to EUnetHTA, other initiatives have also emerged to promote collaboration between countries and regions. The Beneluxa collaboration was launched in 2015 by the Netherlands and Belgium, and since then Luxembourg, Austria, and Ireland have joined. This initiative aims to ensure sustainable access to innovative medicine at affordable costs. Beneluxa encompasses joint horizon scanning, joint writing and mutual recognition of HTA assessments, information sharing, and collaboration on price negotiations (79). Other national initiatives, such as the Danish Medicines Council, have also emerged to ensure fast and homogeneous use of expensive treatments across hospitals and geographical regions in Denmark (80).

Our study has several limitations. First, we only included the assessments of AD drugs and therefore our findings on regulatory and HTA alignment may not fully apply to other disease areas. Furthermore, since no new AD drugs were approved recently, this study only included assessments of relatively old drugs, whilst regulatory and HTA processes will have evolved in recent years. Lastly, we only included two HTA bodies: NICE and ZiN. A large number of HTA bodies exist in Europe and we did not explore important differences in evidence requirements that might exist between European HTA bodies.

In conclusion, our study shows that in the case of established AD, evidence requirements of regulatory and HTA assessments were not that far apart. There was a large overlap in inclusion of phase III trials and use of outcome domains, although the focus on specific outcomes slightly differed. Further alignment might be possible if regulatory authorities use the totality of evidence, including secondary endpoints, explicitly in their benefit-risk assessments and anticipate on collecting real world data to monitor drugs over their life-cycle. New challenges will arise with disease-modifying AD drugs that are currently in development and no gold standard for efficacy measures have been established for these patient groups. These drugs will target people before showing symptoms or when they only experience mild cognitive impairment. Parallel scientific advice and regulatory-HTA cross-talk can facilitate alignment of regulatory and HTA evidence requirements when informed with data that provide evolving insight in regulatory decisions from the past.

## Author Contributions

All authors contributed substantially to the conception and design of the work. MD, AM, and DO collected the data. MD, JB, DO, PJ, and CG were involved in the interpretation of the data. MD drafted the work and the remainder of the authors (JB, DO, RT, AM, PJ, and CG) revised the manuscript critically. All authors read and approved the final version of the manuscript.

### Conflict of Interest Statement

RT is employed by Biogen, Switzerland. The remaining authors declare that the research was conducted in the absence of any commercial or financial relationships that could be construed as a potential conflict of interest.

## References

[1] Launer LJ Andersen K Dewey ME Letenneur L Ott A Amaducci LA. Rates and risk factors for dementia and Alzheimer's disease: results from EURODEM pooled analyses. EURODEM incidence research group and work groups. european studies of dementia Neurology. (1999) 52:78–84. 10.1212/WNL.52.1.78 9921852

[2] Tayeb HO Yang HD Price BH Tarazi FI. Pharmacotherapies for Alzheimer's disease: beyond cholinesterase inhibitors. Pharmacol Ther. (2012) 134:8–25. 10.1016/j.pharmthera.2011.12.002 22198801

[3] Cummings J Lee G Mortsdorf T Ritter A Zhong K. Alzheimer's disease drug development pipeline: 2017. Alzheimers Dement. (2017) 3:367–84. 10.1016/j.trci.2017.05.002 29067343 PMC5651419

[4] European Medicines Agency Available online at: https://www.ema.europa.eu/about-us/what-we-do/authorisation-medicines. (accessed October 26, 2018).

[5] National Institute of Health and Care Excellence Available online at: https://www.nice.org.uk/about/what-we-do/our-programmes/nice-guidance/nice-technology-appraisal-guidance. (accessed October 26, 2018).

[6] De Boer JE Pasman P Procedure Beoordeling Extramurale Geneesmiddelen. Available inline at: https://www.zorginstituutnederland.nl/publicaties/rapport/2016/09/09/procedure-beoordeling-extramurale-geneesmiddelen. Septemer 9, 2016. (accessed October 26, 2018).

[7] Ciani O Jommi C. The role of health technology assessment bodies in shaping drug development. Drug Des Devel Ther. (2014) 8:2273–81. 10.2147/DDDT.S49935 25419117 PMC4234281

[8] European Medicines Agency Available online at: https://www.ema.europa.eu/human-regulatory/research-development/scientific-advice-protocol-assistance/parallel-consultation-regulators-health-technology-assessment-bodies. (accessed October 26, 2018).

[9] European Medicines Agency Available online at: https://www.ema.europa.eu/medicines/human/EPAR/exelon. (accessed October 29, 2018).

[10] European Medicines Agency Available online at: https://www.ema.europa.eu/medicines/human/EPAR/ebixa. (accessed October 29, 2018).

[11] Van der Graaff M Donepezil (hydrochloride) (Aspen) *bij symptomatische behandeling van licht tot matig ernstige dementie van het Alzheimertype*. Available online at: https://www.zorginstituutnederland.nl/publicaties/adviezen/2013/06/24/donepezil-hydrochloride-aspen-bij-symptomatische-behandeling-van-licht-tot-matig-ernstige-dementie-van-het-alzheimertype.pdf. (accessed July 8, 2013).

[12] Rogers SL Farlow MR Doody RS Mohs R Friedhoff LT. A 24-week, double-blind, placebo-controlled trial of donepezil in patients with Alzheimer's disease. donepezil study group. Neurology. (1998) 50:136–45. 10.1212/WNL.50.1.136 9443470

[13] Rogers SL Doody RS Mohs RC Friedhoff LT. Donepezil improves cognition and global function in Alzheimer disease: a 15-week, double-blind, placebo-controlled study. Donepezil Study Group. Arch Intern Med. (1998) 158:1021–31. 10.1001/archinte.158.9.1021 9588436

[14] Burns A Rossor M Hecker J Gauthier S Petit H Moller HJ. The effects of donepezil in Alzheimer's disease - results from a multinational trial. Dement Geriatr Cogn Disord. (1999) 10:237–44. 10.1159/000017126 10325453

[15] Courtney C Farrell D Gray R Hills R Lynch L Sellwood E. Long-term donepezil treatment in 565 patients with Alzheimer's disease (AD2000): randomised double-blind trial. Lancet. (2004) 363:2105–15. 10.1016/S0140-6736(04)16499-4 15220031

[16] Gauthier S Feldman H Hecker J Vellas B Emir B Subbiah P. Functional, cognitive and behavioral effects of donepezil in patients with moderate Alzheimer's disease. Curr Med Res Opin. (2002) 18:347–54. 10.1185/030079902125001029 12442882

[17] Greenberg SM Tennis MK Brown LB Gomez-Isla T Hayden DL Schoenfeld DA. Donepezil therapy in clinical practice: a randomized crossover study. Arch Neurol. (2000) 57:94–9. 10.1001/archneur.57.1.94 10634454

[18] Holmes C Wilkinson D Dean C Vethanayagam S Olivieri S Langley A. The efficacy of donepezil in the treatment of neuropsychiatric symptoms in Alzheimer disease. Neurology. (2004) 63:214–9. 10.1212/01.WNL.0000129990.32253.7B 15277611

[19] Homma A Takeda M Imai Y Udaka F Hasegawa K Kameyama M. Clinical efficacy and safety of donepezil on cognitive and global function in patients with Alzheimer's disease. a 24-week, multicenter, double-blind, placebo-controlled study in Japan. E2020 study group. Dement Geriatr Cogn Disord. (2000) 11:299–313. 10.1159/000017259 11044775

[20] Johannsen P Salmon E Hampel H Xu Y Richardson S Qvitzau S. Assessing therapeutic efficacy in a progressive disease: a study of donepezil in Alzheimer's disease. CNS Drugs. (2006) 20:311–25. 10.2165/00023210-200620040-00005 16599649

[21] Krishnan KR Charles HC Doraiswamy PM Mintzer J Weisler R Yu X. Randomized, placebo-controlled trial of the effects of donepezil on neuronal markers and hippocampal volumes in Alzheimer's disease. Am J Psychiatry. (2003) 160:2003–11. 10.1176/appi.ajp.160.11.2003 14594748

[22] Mazza M Capuano A Bria P Mazza S. Ginkgo biloba and donepezil: a comparison in the treatment of Alzheimer's dementia in a randomized placebo-controlled double-blind study. Eur J Neurol. (2006) 13:981–5. 10.1111/j.1468-1331.2006.01409.x 16930364

[23] Mohs RC Doody RS Morris JC Ieni JR Rogers SL Perdomo CA. A 1-year, placebo-controlled preservation of function survival study of donepezil in AD patients. Neurology. (2001) 57:481–8. 10.1212/WNL.57.3.481 11502917

[24] Moraes W Poyares D Sukys-Claudino L Guilleminault C Tufik S. Donepezil improves obstructive sleep apnea in Alzheimer disease: a double-blind, placebo-controlled study. Chest. (2008) 133:677–83. 10.1378/chest.07-1446 18198262

[25] Moraes Wdos S Poyares DR Guilleminault C Ramos LR Bertolucci PH Tufik S. The effect of donepezil on sleep and REM sleep EEG in patients with Alzheimer disease: a double-blind placebo-controlled study. Sleep. (2006) 29:199–205. 10.1093/sleep/29.2.199 16494088

[26] Peng DT Xu XH Wang LN Efficiency and safety assessment of donepezil for treating mild and moderate Alzheimer disease. Chinese J Clin Rehab. (2005) 9:170–72.

[27] Rogers SL Friedhoff LT. The efficacy and safety of donepezil in patients with Alzheimer's disease: results of a US multicentre, randomized, double-blind, placebo-controlled trial. the donepezil study group. Dementia. (1996) 7:293–303. 8915035 10.1159/000106895

[28] Seltzer B Zolnouni P Nunez M Goldman R Kumar D Ieni J. Efficacy of donepezil in early-stage Alzheimer disease: a randomized placebo-controlled trial. Arch Neurol. (2004) 61:1852–6. 10.1001/archneur.61.12.1852 15596605

[29] Winblad B Engedal K Soininen H Verhey F Waldemar G Wimo A A 1-year, randomized, placebo-controlled study of donepezil in patients with mild to moderate AD. Neurology. (2001) 57:489–95. 10.1212/WNL.57.3.489 11502918

[30] Winstein CJ Bentzen KR Boyd L Schneider LS. Does the cholinesterase inhibitor, donepezil, benefit both declarative and non-declarative processes in mild to moderate Alzheimer's disease? Curr Alzheimer Res. (2007) 4:273–6. 10.2174/156720507781077296 17627484

[31] Bullock R Touchon J Bergman H Gambina G He Y Rapatz G. Rivastigmine and donepezil treatment in moderate to moderately-severe Alzheimer's disease over a 2-year period. Curr Med Res Opin. (2005) 21:1317–27. 10.1185/030079905X56565 16083542

[32] Ancoli-Israel S Amatniek J Ascher S Sadik K Ramaswamy K. Effects of galantamine versus donepezil on sleep in patients with mild to moderate Alzheimer disease and their caregivers: a double-blind, head-to-head, randomized pilot study. Alzheimer Dis Assoc Disord. (2005) 19:240–5. 10.1097/01.wad.0000189052.48688.36 16327351

[33] Cumbo E Differential effects of rivastigmine, galantamine and donepezil on behavioral and psychological symptoms in patients with Alzheimer's disease: 18-month, randomized, open-label trial. Prim Care Commu Psychiatry. (2005) 10:95–102. 10.1185/135525705X40436

[34] Fuschillo C La Pia S Campana F Pinto A De Simone L. Cognitive deficits in Alzheimer's disease: treatment with acetylcholinesterase inhibitor agents. Arch Gerontol Geriatr Suppl. (2001) 7:151–8. 10.1016/S0167-4943(01)00134-0 11431059

[35] Jones RW Soininen H Hager K Aarsland D Passmore P Murthy A. A multinational, randomised, 12-week study comparing the effects of donepezil and galantamine in patients with mild to moderate Alzheimer's disease. Int J Geriatr Psychiatry. (2004) 19:58–67. 10.1002/gps.1038 14716700

[36] Nordberg A Darreh-Shori T Peskind E Soininen H Mousavi M Eagle G. Different cholinesterase inhibitor effects on CSF cholinesterases in Alzheimer patients. Curr Alzheimer Res. (2009) 6:4–14. 10.2174/156720509787313961 19199870 PMC4046577

[37] Wilkinson DG Passmore AP Bullock R Hopker SW Smith R Potocnik FC. A multinational, randomised, 12-week, comparative study of donepezil and rivastigmine in patients with mild to moderate Alzheimer's disease. Int J Clin Pract. (2002) 56:441–6. 12166542

[38] Hansen RA Gartlehner G Lohr KN Kaufer DI. Functional outcomes of drug treatment in Alzheimer's disease: a systematic review and meta-analysis. Drugs Aging. (2007) 24:155–67. 10.2165/00002512-200724020-00007 17313203

[39] German Institute for Quality and Efficiency in Health Care Cholinesterase inhibitors in Alzheimer's disease. Available online at: https://www.iqwig.de/. (accessed February 2007).

[40] Hansen RA Gartlehner G Webb AP Morgan LC Moore CG Jonas DE. Efficacy and safety of donepezil, galantamine, and rivastigmine for the treatment of Alzheimer's disease: a systematic review and meta-analysis. Clin Interv Aging. (2008) 3:211–25. 18686744 PMC2546466

[41] Wilcock G Howe I Coles H Lilienfeld S Truyen L Zhu Y. A long-term comparison of galantamine and donepezil in the treatment of Alzheimer's disease. Drugs Aging. (2003) 20:777–89. 10.2165/00002512-200320100-00006 12875613

[42] Schneider LS Anand R Farlow MR Systematic review of the efficacy of rivastigmine for patients with Alzheimer's disease. Int J Geriatric Psychopharmacol. (1998) 1:S26–34.

[43] Corey-Bloom J Anand R Veach J for the ENA 713 B352 Study Group A randomized trial evaluating the efficacy and safety of ENA 713 (rivastigmine tartrate), a new acetylcholinesterase inhibitor, in patients with mild to moderately sever Alzheimer's disease. Int J Geriatric Psychopharmacol. (1998) 1:55–65.

[44] Rosler M Anand R Cicin-Sain A Gauthier S Agid Y Dal-Bianco P. Efficacy and safety of rivastigmine in patients with Alzheimer's disease: international randomised controlled trial. BMJ. (1999) 318:633–8. 10.1136/bmj.318.7184.633 10066203 PMC27767

[45] Feldman HH Lane R Study G. Rivastigmine: a placebo controlled trial of twice daily and three times daily regimens in patients with Alzheimer's disease. J Neurol Neurosurg Psychiatry. (2007) 78:1056–63. 10.1136/jnnp.2006.099424 17353259 PMC2117538

[46] Agid YD Anand B Gharabawi G Efficacy and tolerability of rivastigmine in patients with dementia of the Alzheimer type. Curr Ther Res. (1998) 59:837–45. 10.1016/S0011-393X(98)85048-0

[47] Forette F Anand R Gharabawi G. A phase II study in patients with Alzheimer's disease to assess the preliminary efficacy and maximum tolerated dose of rivastigmine (Exelon). Eur J Neurol. (1999) 6:423–9. 10.1046/j.1468-1331.1999.640423.x 10362894

[48] Mowla A Mosavinasab M Haghshenas H Borhani Haghighi A. Does serotonin augmentation have any effect on cognition and activities of daily living in Alzheimer's dementia? a double-blind, placebo-controlled clinical trial. J Clin Psychopharmacol. (2007) 27:484–7. 10.1097/jcp.0b013e31814b98c1 17873681

[49] Winblad B Cummings J Andreasen N Grossberg G Onofrj M Sadowsky C. A six-month double-blind, randomized, placebo-controlled study of a transdermal patch in Alzheimer's disease–rivastigmine patch versus capsule. Int J Geriatr Psychiatry. (2007) 22:456–67. 10.1002/gps.1788 17380489

[50] Birks J Grimley Evans J Iakovidou V Tsolaki M Rivastigmine for Alzheimer's disease. Cochrane Database Syst Rev. (2000) CD001191. 10.1002/14651858.CD001191 11034705

[51] Wilcock GK Lilienfeld S Gaens E. Efficacy and safety of galantamine in patients with mild to moderate Alzheimer's disease: multicentre randomised controlled trial. galantamine international-1 study group. BMJ. (2000) 321:1445–9. 10.1136/bmj.321.7274.1445 11110737 PMC27547

[52] Rockwood K Mintzer J Truyen L Wessel T Wilkinson D. Effects of a flexible galantamine dose in Alzheimer's disease: a randomised, controlled trial. J Neurol Neurosurg Psychiatry. (2001) 71:589–95. 10.1136/jnnp.71.5.589 11606667 PMC1737604

[53] Raskind MA Peskind ER Wessel T Yuan W. Galantamine in AD: A 6-month randomized, placebo-controlled trial with a 6-month extension. the galantamine USA-1 study group. Neurology. (2000) 54:2261–8. 10.1212/WNL.54.12.2261 10881250

[54] Tariot PN Solomon PR Morris JC Kershaw P Lilienfeld S Ding C. A 5-month, randomized, placebo-controlled trial of galantamine in AD. the galantamine USA-10 study group. Neurology. (2000) 54:2269–76. 10.1212/WNL.54.12.2269 10881251

[55] Wilkinson D Murray J. Galantamine: a randomized, double-blind, dose comparison in patients with Alzheimer's disease. Int J Geriatr Psychiatry. (2001) 16:852–7. 10.1002/gps.409 11571763

[56] Bullock R Erkinjuntti T Lilienfeld S GAL-INT-6 Study Group. Management of patients with Alzheimer's disease plus cerebrovascular disease: 12-month treatment with galantamine. Dement Geriatr Cogn Disord. (2004) 17:29–34. 10.1159/000074140 14560062

[57] Brodaty H Corey-Bloom J Potocnik FC Truyen L Gold M Damaraju CR. Galantamine prolonged-release formulation in the treatment of mild to moderate Alzheimer's disease. Dement Geriatr Cogn Disord. (2005) 20:120–32. 10.1159/000086613 15990426

[58] Cummings JL Schneider L Tariot PN Kershaw PR Yuan W. Reduction of behavioral disturbances and caregiver distress by galantamine in patients with Alzheimer's disease. Am J Psychiatry. (2004) 161:532–8. 10.1176/appi.ajp.161.3.532 14992980

[59] Rockwood K Fay S Song X MacKnight C Gorman M Video-Imaging Synthesis of Treating Alzheimer's Disease (VISTA) Investigators. Attainment of treatment goals by people with Alzheimer's disease receiving galantamine: a randomized controlled trial. CMAJ. (2006) 174:1099–105. 10.1503/cmaj.051432 16554498 PMC1421447

[60] Erkinjuntti T Kurz A Gauthier S Bullock R Lilienfeld S Damaraju CV. Efficacy of galantamine in probable vascular dementia and Alzheimer's disease combined with cerebrovascular disease: a randomised trial. Lancet. (2002) 359:1283–90. 10.1016/S0140-6736(02)08267-3 11965273

[61] Olin JT Schneider L Galantamine for Alzheimer's disease. Cochrane Database Syst Rev. (2002) CD001747. 10.1002/14651858.CD001747 12137632

[62] NICE Guidance on the use of donepezil, rivastigmine, and galantamine for the treatment of Alzheimer's disease. Techonology appraisal guidance 19 2001.

[63] Reisberg B Doody R Stoffler A Schmitt F Ferris S Mobius HJ Memantine study G. memantine in moderate-to-severe Alzheimer's disease. N Engl J Med. (2003) 348:1333–41. 10.1056/NEJMoa013128 12672860

[64] Winblad B Poritis N. Memantine in severe dementia: results of the 9M-best study (Benefit and efficacy in severely demented patients during treatment with memantine). Int J Geriatr Psychiatry. (1999) 14:135–46. 10.1002/(SICI)1099-1166(199902)14:2<135::AID-GPS906>3.0.CO;2-0 10885864

[65] Wilcock G Mobius HJ Stoffler A MMM 500 group. A double-blind, placebo-controlled multicentre study of memantine in mild to moderate vascular dementia (MMM500). Int Clin Psychopharmacol. (2002) 17:297–305. 10.1097/00004850-200211000-00005 12409683

[66] Orgogozo JM Rigaud AS Stoffler A Mobius HJ Forette F. Efficacy and safety of memantine in patients with mild to moderate vascular dementia: a randomized, placebo-controlled trial (MMM 300). Stroke. (2002) 33:1834–9. 10.1161/01.STR.0000020094.08790.49 12105362

[67] van Dyck CH Tariot PN Meyers B Malca Resnick E Memantine MEMMDSG. a 24-week randomized, controlled trial of memantine in patients with moderate-to-severe Alzheimer disease. Alzheimer Dis Assoc Disord. (2007) 21:136–43. 10.1097/WAD.0b013e318065c495 17545739

[68] Tariot PN Farlow MR Grossberg GT Graham SM McDonald S Gergel I. Memantine treatment in patients with moderate to severe Alzheimer disease already receiving donepezil: a randomized controlled trial. JAMA. (2004) 291:317–24. 10.1001/jama.291.3.317 14734594

[69] Doody R Wirth Y Schmitt F Mobius HJ. Specific functional effects of memantine treatment in patients with moderate to severe Alzheimer's disease. Dement Geriatr Cogn Disord. (2004) 18:227–32. 10.1159/000079833 15256834

[70] Rive B Vercelletto M Damier FD Cochran J Francois C. Memantine enhances autonomy in moderate to severe Alzheimer's disease. Int J Geriatr Psychiatry. (2004) 19:458–64. 10.1002/gps.1112 15156547

[71] Porsteinsson AP Grossberg GT Mintzer J Olin JT. Memantine treatment in patients with mild to moderate Alzheime's disease already receiving a cholinesterase inhibitor: a randomized, double-blind, placebo-controlled trial. Curr Alzheimer Res. (2008) 5:83–89. 10.2174/156720508783884576 18288936

[72] Peninsula Technology Assessment Group (PenTAG) The Effectiveness and Cost-Effectiveness of Donepezil, Galantamine, Rivastigmine and Memantine for the Treatment of Alzheimer's Disease (review of TA11)1: A Systematic Review and Economic Model. Available online at: https://www.nice.org.uk/guidance/ta217/documents/alzheimers-disease-donepezil-galantamine-rivastigmine-and-memantine-review-assessment-report-part-12. (accessed June 18, 2010). 10.3310/hta16210 PMC4780923 22541366

[73] Clegg A Bryant J Nicholson T McIntyre L De Broe S Gerard K. Clinical and cost-effectiveness of donepezil, of donepezil, rivastigmine and galantamine for Alzheimer's disease. A systematic review. Int JTechnol Assess Health Care. (2002) 18:497–507. 10.1017/S026646230200034X 12391943

[74] European Medicines Agency Committee for Medicinal Products for Human Use Guideline on the clinical investigation of medicines for the treatment of Alzheimer's disease. Available online at: https://www.ema.europa.eu/documents/scientific-guideline/guideline-clinical-investigation-medicines-treatment-alzheimers-disease-revision-2_en.pdf. Published February 22, 2018. (accessed October 29, 2018).

[75] European Medicines Agency Guideline on good pharmacovigilance practices (GVP). Module VII - Periodic safety update report. Available online at: https://www.ema.europa.eu/documents/scientific-guideline/guideline-good-pharmacovigilance-practices-gvp-module-vii-periodic-safety-update-report_en.pdf. Published July 2, 2012. First update December 9, 2013. (accessed October 29, 2018).

[76] European Medicines Agency Available online at: https://www.ema.europa.eu/human-regulatory/research-development/prime-priority-medicines. (accessed October 29, 2018).

[77] Wang T McAuslane N Liberti L Leufkens H Hovels A. Building synergy between regulatory and HTA agencies beyond processes and procedures-can we effectively align the evidentiary requirements? a survey of stakeholder perceptions. Value Health. (2018) 21:707–14. 10.1016/j.jval.2017.11.003 29909876

[78] Allen A Liberti L Walker SR Salek S. A comparison of reimbursement recommendations by European HTA agencies: is there opportunity for further alignment? Front Pharmacol. (2017) 8:384. 10.3389/fphar.2017.00384 28713265 PMC5491965

[79] Beneluxa Available online at: http://www.beneluxa.org/. (accessed March 21, 2019).

[80] Havermann MC Aagaard L The danish medicines council: a new prioritisation organ for medicine use in hospitals. Eur Pharm Law Review. (2018) 2:85–89. 10.21552/eplr/2018/2/6

