# Supplementary material for: Alignment of European Regulatory and Health Technology Assessments: A Review of Licensed Products for Alzheimer's Disease
Source: Front Med (Lausanne). 2019 May 7;6:73. doi: 10.3389/fmed.2019.00073 (PMC6515927; doi:10.3389/fmed.2019.00073)
Supplement: Supplementary file 1 [file Table_1.docx]

**Supplementary Table 1**. Characteristics of pivotal trials of the marketing authorisation application dossiers of AD medicinal products.

| **Drug / Manufacturer** | **Trial** | **Trial arms** | **Number of participants** | **Primary outcome measure** | **Secondary outcome measure** | **Types of AD, and severity of disease** |
| --- | --- | --- | --- | --- | --- | --- |
| **Donepezil** | E2020-A0001-302 [12]* | 5 mg/day  10 mg/day  Placebo | 582  588  589 | ADAS-cog  CIBIC-plus | MMSE  CDR-SB  QoL | Probable mild-to moderate AD |
|  | E2020-A0001-301 [13] | 5 mg/day  10 mg/day  Placebo | 154  157  162 | ADAS-cog  CIBIC-plus | MMSE  CDR-SB  QoL | Probable mild-to moderate AD |
|  | E2020-A0001-304 [14] | 5 mg/day  10 mg/day  Placebo | 271  273  274 | ADAS-cog  CIBIC-plus | CDR-SB  QoL  IDDD | Probable mild-to moderate AD |
| **Rivastigmine** | B351 [42] | 3 mg/day  6 mg/day  9mg/day  Placebo | 175  176  178  173 | ADAS-cog  CIBIC-plus | PDS  MMSE  GDS | Probable mild-to-moderate AD |
|  | B352 [43] | 1-4 mg/day  6-12 mg/day  Placebo | 233  231  235 | ADAS-cog  CIBIC-plus | PDS  MMSE  GDS | Probable mild-to-moderate AD |
|  | B303 [44] | 1-4 mg/day  6-12 mg/day  Placebo | 243  243  239 | ADAS-cog  CIBIC-plus | PDS  MMSE  GDS | Probable mild-to-moderate AD |
|  | B304 [45] | 2-12 mg BID  2-12 mg TID  Placebo | 118  111  117 | ADAS-cog  CIBIC-plus | CIBIC-Plus  PDS  ADAS-cogA  MMSE  GDS | Probable mild-to-moderate AD |
| **Galantamine** | GAL-95-05 | 24 mg/day  Placebo | 275  279 | ADAS-cog  CIBIC-plus  NOS-GER | MMSE  ADAS-non-cog  DSST  NAB | Probable mild-to moderate AD |
|  | GAL-INT-1 [51] | 24 mg/day  32 mg/day  Placebo | 220  218  215 | ADAS-cog/11  CIBIC-plus | DAD | Probable mild-to moderate AD |
|  | GAL-INT-2 [52] | 24 or 32 mg/day  Placebo | 261  125 | ADAS-cog/11  CIBIC-plus | DAD  NPI | Probable mild-to moderate AD |
|  | GAL-USA-1 [53] | 24 mg/day  32 mg/day  Placebo | 212  211  213 | ADAS-cog/11  CIBIC-plus | DAD | Probable mild-to-moderate AD |
|  | GAL-USA-10 [54] | 8 mg/day  16 mg/day  24 mg/day  Placebo | 140  279  273  286 | ADAS-cog/11  CIBIC-plus | ADCS-ADL  NPI | Probable mild-to-moderate AD |
| **Memantine** | MRZ-9605 [63] | 20 mg/daily  Placebo | 126  126 | CIBIC-plus  ADCS-ADLsev | SIB  MMSE  FAST  GDS  NPI | Probable moderate-to-severe AD |
|  | MRZ-9403 [64] | 10 mg/day  Placebo | 82  84 | CGI-C  BGP, subscore dependence | Modified D-scale  CGI-S  BGP total score | Probable moderate-to-severe dementia of AD, vascular or mixed type |
|  | MRZ-9202 [65] | 20 mg/day  Placebo | 295  284 | ADAS-cog  CGI-C | GBS  NOS-GER  MMSE | Probable moderate-to-severe vascular dementia |
|  | MRZ-9408 [66] | 20 mg/day  Placebo | 165  156 | ADAS-cog  CIBIC-plus | MMSE  GBS  CGI-C  NOS-GER | Probable mild-to-moderate vascular dementia |

* The reference numbers correspond to the reference list of the main article. ADAS-cog Alzheimer's Disease Assessment Scale-Cognitive Subscale, CIBIC-plus Clinician's Interview-Based Impression of Change Plus Caregiver Input, CDR-SB Clinical Dementia Rating sum of Boxes, QoL Quality of life, IDDD Interview for Deterioration of Daily Living Activities in Dementia, ADAS-non-cog Alzheimer's Disease Assessment Scale-Noncognitive, DDST Digit Symbol Substitution Test, NAB Nürnbergers-Alters Beobachtungs-Skala, ADAS-cog/11 11 item cognitive subscale of the ADAS-cog, DAD disability Assessment in Dementia, ADCS-ADL Alzheimer’s Disease Cooperative Study-Activities of Daily Living Inventory, PDS progressive deterioration scale, MMSE Mini-Mental State Exam, GDS global deterioration scale, ADAS-cogA ADAS-cog with added item of attention (concentration/distractibility) , ADCS-ADLsev Alzheimer’s Disease Cooperative Study-Activities of Daily Living Inventory modified for severe dementia, FAST Functional Assessment staging, NPI neuropsychiatric inventory, BGP Behavioural Rating Scale for Geriatric Patients, Modified D-scale: extended and validated version of the D-test, CGI-S Clinical Global Impression of Severity, GBS Gottfries-Brane-Steen scale, CGI-C Clinical Global Impression of Change, NOS-GER Nurses' Observation Scale for Geriatric Patients, SIB severe impairment battery.
