# Supplementary material for: Alignment of European Regulatory and Health Technology Assessments: A Review of Licensed Products for Alzheimer's Disease
Source: Front Med (Lausanne). 2019 May 7;6:73. doi: 10.3389/fmed.2019.00073 (PMC6515927; doi:10.3389/fmed.2019.00073)
Supplement: Supplementary file 2 [file Table_2.docx]

**Supplementary Table 2.** Glossary of different outcome measures per outcome domain.

| **Outcome domain** | **Outcome measure per outcome domain** |
| --- | --- |
| Cognition | **ADAS-cog:** neuropsychological test consisting of an 11-item scale used to assess the severity of cognitive impairment in the following areas: memory, language, orientation, reason, and praxis  **MMSE:** a 30-point questionnaire that measures cognitive impairment  **SIB:** neuropsychological test consisting of 40 items divided across the following nine cognitive domains: language, memory, praxis, orientation, orienting to name, construction, social interaction, attention, visuospatial ability  **DSST:** a neuropsychological test in which the subject has to assign the correct symbol to digits ranging from 1 to 9 according to a code table displaying pairs of digits and symbols in a 90 seconds time period. This task requires response speed, sustained attention, visual spatial skills and set shifting. |
| Global effect | **CIBIC-plus:** global rating score that reflects patient function in four areas: general, cognitive, behaviour, and activities of daily living  **NOS-GER:** rating scale that rates the daily behavior of elderly patients and measures impairment in the following six areas: memory, instrumental activities of daily living, (basic) activities of daily living, mood, social behavior, and disturbing behavior  **CDR-SB:** clinical measure that covers the domains memory, orientation, judgment and problem solving, community affairs, home and hobbies, and personal care  **GDS:** stages deficits in cognition and function and describes seven stages of dementia from normal functioning to severe dementia  **GBS:** observer scale that consists of three subscales measuring impairment of motor performance, impairment of intellectual and emotional capacity, and six individual symptoms commonly observed in dementia  **CGI-S:** 7-point scale that requires the clinician to rate the [severity of the patient's illness](https://en.wikipedia.org/wiki/Severity_of_illness) at the time of assessment, relative to the clinician's past experience with patients who have the same [diagnosis](https://en.wikipedia.org/wiki/Medical_diagnosis)  **CGI-C:** 7-point scale that requires the [clinician](https://en.wikipedia.org/wiki/Clinician) to assess how much the patient's illness has improved or worsened relative to a baseline state at the beginning of the intervention  **BGP:** 35-item observer scale for the assessment of functional and behavioural disturbances of geriatric patients. The BGP contains the following four subscales: behaviour, cognition, activities of daily living, and mobility |
| Function | **IDDD:** 33-item caregiver-based measure for the assessment of the initiative to perform and actual performance of self-care and more complex activities  **DAD**: 40-item caregiver-based scale that evaluates the basic and instrumental activities of daily living in a wide range of functional domains: eating, meal preparation, telephoning, hygienic, dressing, medication, corresponding, finance, leisure, and housework  **FAST:** assesses the magnitude of progressive functional deterioration in patients with dementia by identifying characteristic progressive disabilities. Its seven major stages range from normal to severe dementia.  **PDS:** 29-item scale for caregivers that examines the ability of patients to accomplish basic ADLs and IADLs in 11 areas  **ADCS-ADL:**  structured caregiver based interview to assess ADL. The 19-item version (ADCS-ADL_19_), covering mainly basic ADL, is used for the assessment of patients with more severe AD, while the 23-item version (ADCS-ADL_23_) includes more complex ADL for the assessment of mild to moderate AD, such as reading books or magazines, pastime activities, or household chores  **NAB:** rating-scale validated for German-speaking countries, which assesses the degree of independence or care dependency by means of the patient’s ability or inability to cope with everyday tasks  **Modified D-scale:** Ferm's D-test evaluates behavioural activities and functioning. The modified D-scale is an extended version with sixteen items that assess independence, ranging from normal functioning to total care dependence.  **BADLS:** 20-item caregiver-based questionnaire designed to measure the ability of to carry out daily activities such as dressing, preparing food and using transport. |
| Behaviour and mood | **NPI:** examines 10 sub-domains of behavioral functioning: delusions, hallucinations, agitation/aggression, dysphoria, anxiety, euphoria, apathy, disinhibition, irritability/lability, and aberrant motor activity. Two other sub-domains have been added since its development: night-time behavioral disturbances and appetite and eating abnormalities. |
| Quality of life | **EQ-5D:** a standardised 5-item instrument that evaluates mobility, self-care, mood, daily activities, and physical complaints /pain. The questionnaire can be used as an index to measure a utility value.  **Qol:** patient rated seven-item scale that evaluates the patient’s feeling of well-being |

ADAS-cog Alzheimer's Disease Assessment Scale- Cognitive Subscale, MMSE Mini-Mental State Exam, SIB severe impairment battery, DDST Digit Symbol Substitution Test, CIBIC-plus Clinician's Interview-Based Impression of Change Plus Caregiver Input, Nurses' Observation Scale for Geriatric Patients, CDR-SB Clinical Dementia Rating sum of Boxes, GDS global deterioration scale, GBS Gottfries-Brane-Steen scale, CGI-S Clinical Global Impression of Severity, CGI-C Clinical Global Impression of Change, BGP Behavioural Rating Scale for Geriatric Patients, IDDD Interview for Deterioration of Daily Living Activities in Dementia, DAD disability Assessment in Dementia, FAST Functional Assessment staging, PDS progressive deterioration scale, ADCS-ADL Alzheimer’s Disease Cooperative Study-Activities of Daily Living Inventory, NAB Nürnbergers-Alters Beobachtungs-Skala, Modified D-scale: extended and validated version of the D-test, BADLS Bristol Activities of Daily Living scale, NPI neuropsychiatric inventory, QoL Quality of life.
